# Analysis of Pregnancy-Associated Plasma Protein A Production in Human Adult Cardiac Progenitor Cells

**DOI:** 10.1155/2013/190178

**Published:** 2013-11-10

**Authors:** Piera D'Elia, Vittoria Ionta, Isotta Chimenti, Francesco Angelini, Fabio Miraldi, Alessandro Pala, Elisa Messina, Alessandro Giacomello

**Affiliations:** ^1^Department of Gynaecology, Obstetrics and Urologic Sciences, Sapienza University of Rome, Viale Regina Elena 324, 00161 Rome, Italy; ^2^Department of Molecular Medicine, Cenci Bolognetti Foundation, Pasteur Institute, Sapienza University of Rome, Viale Regina Elena 324, 00161 Rome, Italy; ^3^Department of Medical Surgical Sciences and Biotechnology, Sapienza University of Rome, Corso della Repubblica 79, 04100 Latina, Italy; ^4^Department of Cardiocirculatory Pathophysiology, Anesthesiology and General Surgery, Sapienza University of Rome, Viale Regina Elena 324, 00161 Rome, Italy

## Abstract

IGF-binding proteins (IGFBPs) and their proteases regulate IGFs bioavailability in multiple tissues. Pregnancy-associated plasma protein A (PAPP-A) is a protease acting by cleaving IGFBP2, 4, and 5, regulating local bioavailability of IGFs. We have previously shown that IGFs and IGFBPs are produced by human adult cardiac progenitor cells (haCPCs) and that IGF-1 exerts paracrine therapeutic effects in cardiac cell therapy with CPCs. Using immunofluorescence and enzyme immunoassays, we firstly report that PAPP-A is produced and secreted in surprisingly high amounts by haCPCs. In particular, the homodimeric, enzymatically active, PAPP-A is secreted in relevant concentrations in haCPC-conditioned media, while the enzymatically inactive PAPPA/proMBP complex is not detectable in the same media. Furthermore, we show that both homodimeric PAPP-A and proMBP can be detected as cell associated, suggesting that the previously described complex formation at the cell surface does not occur easily, thus positively affecting IGF signalling. Therefore, our results strongly support the importance of PAPP-A for the IGFs/IGFBPs/PAPP-A axis in CPCs biology.

## 1. Introduction

IGFs, IGF receptors, and IGF-binding proteins (IGFBPs) are expressed in the heart, being involved in tissue homeostasis, and their levels change locally following infarction [1], participating in postischaemic neovascularization and stimulating the reentry of adult ventricular myocytes into the cell cycle [2]. Interaction of IGF-1 with its receptor stimulates DNA and protein synthesis and contractility and inhibits apoptosis in cardiomyocytes. IGF-1 is also robustly released by heart-biopsy-derived cardiac progenitors cells (CPCs) cultured *ex vivo *as cardiospheres (CSps) [3] and CSp-derived cells (CDCs) [4], contributing to their autocrine and paracrine function [5] in cardiac regenerative medicine approaches [6, 7] of cell therapy [8] and tissue engineering [9, 10]. Furthermore, IGF-1 is produced by cardiac fibroblasts [11] and has a regulatory role in the stem cell niche [12].

At tissue level, IGFBP proteases control IGFs availability, and they are individually regulated by specific inhibitors. The most fully characterized is the pregnancy-associated plasma protein A (PAPP-A) system for cleaving IGFBPs. IGFBPs bind IGFs with similar or higher affinities than IGF-receptors, regulating their biological activities by acting as a reservoir of IGFs in the circulation and by modulating the interactions between IGFs and their receptors at cellular level. PAPP-A exerts a specific proteolytic activity on IGFBP-4 [13], IGFBP-5 [14], and IGFBP-2 [15], thus directly contributing to the extent of IGF-dependent cellular effects. The widespread distribution of PAPP-A has been also associated with tissue regeneration mechanisms by releasing IGFs that are capable of activating macrophages, stimulating fibroblasts proliferation and matrix protein biosynthesis [16].

PAPP-A is mainly produced not only by the trophoblast during pregnancy (low levels in the first trimester are associated with chromosomal abnormalities [17] and pregnancy-induced hypertension [18]), but also by granulose cells, fibroblasts, osteoblasts, and endothelial and vascular smooth muscle cells [19]. Circulating PAPP-A in pregnancy predominantly exists as a heterotetramer, complexed in a 2 : 2 ratio with its inhibitor, the proform of eosinophil major basic protein (proMBP) [20], and denoted as PAPP-A/proMBP. Elevated serum levels of the active homodimeric PAPP-A (dPAPP-A) have also been found in patients with acute coronary syndrome (ACS). In these cases, pathogenic mechanisms might be due to the increase of local concentration of PAPP-A which, following cleavage of IGFBPs, allows the release and promotion of IGF-related proatherogenic effects [21]. Indeed, it has been shown *in vitro* that IGF is able to induce activation of inflammatory cells and release of inflammatory cytokines by activated macrophages, thus promoting plaque progression and destabilization [21, 22]. Further observations on the role of PAPP-A as mediator of cardiovascular diseases include the association of its serum levels with the severity of heart failure and with the risk for adverse cardiac events [23]. PAPP-A is also emerging as a promising prognostic marker in patients with stable cardiovascular disease [24] and ST-elevation myocardial infarction [25]. 

On the other hand, cardiovascular protective effects of IGF activity have been previously described and include protection against endothelial dysfunction, athererosclerotic plaque development, metabolic syndrome, and ischemic myocardial damage. Interestingly, IGF-1 contributes to endothelial and parenchymal regeneration at the site of tissue damage by expanding the pool of progenitor cells [26]. However, the involvement of PAPP-A in this dynamic system has not been reported and the present study was undertaken to assess the presence and release of PAPP-A by human CPCs, stressing its potential importance on IGFBP modulation and IGF release.

To investigate the balance and regulation of the IGFs/IGFBPs/PAPP-A network in CPCs biology, we assessed the expression and release of PAPP-A forms by CSps and CDCs, using immunological and enzymatic assays specifically detecting dPAPP-A, proMBP, and the PAPP-A/proMBP complex.

## 2. Materials and Methods

### 2.1. Cell Culture

CPCs were isolated and cultured by the CSp method, as previously described [3, 28], from surgical human auricola biopsies, during clinically indicated procedures after informed consent in an institutional review board-approved protocol, conforming with the principles of the Declaration of Helsinki. Briefly, CSps were cultured on poly-D-lysine (BD Biosciences) coated plates at 10^4^ cells/cm^2^ in CSp-growth medium (CGM): 35% IMDM and 65% DMEM/F-12 Mix, 3.5% FBS (Hyclone), 1% penicillin-streptomycin, 1% L-glutamine, 0.1 mmol/L 2-mercaptoethanol, 1 unit/mL thrombin (Sigma), 1 : 50 B-27 (Invitrogen), 80 ng/mL bFGF, 25 ng/mL EGF, and 4 ng/mL cardiotrophin-1 (Peprotech). CDCs were grown on fibronectin (Sigma) coated flasks at 2–4 × 10^4^ cells/cm^2^ in 20% FBS complete explant medium (CEM): IMDM, 1% penicillin-streptomycin, 1% L-glutamine, and 0.1 mM 2-mercaptoethanol. Conditioned media (CMs) were collected after 96 hours. 

### 2.2. Antibodies for Immunocytochemistry (ICC) and Enzyme Immunoassay (EIA)

The following mouse monoclonal antibodies were used for ICC and EIA: antihuman dPAPP-A (4PD4-PAPP2, Hytest, referred to as 4PD4) and antihuman pro-MBP (5H9, Hytest). The following immunoglobulin preparations from antisera were used: rabbit antihuman PAPP-A (A0230, Dako) previously absorbed in a negative affinity chromatography step over an immobilized preparation of pregnancy-specific beta-1-glycoprotein (SP1), obtained as previously described [29]. This latter antibody reacts with both PAPP-A subunits and proMBP and therefore is able to recognize dPAPP-A, PAPP-A/proMBP, and free and complexed proMBP. Alexa Fluor 568 and Alexa Fluor 488 conjugated anti-mouse IgG and anti-rabbit IgG (Invitrogen) were used as secondary antibodies for ICC. Capture antibody used for EIAs was HRP conjugated rabbit antihuman PAPP-A (P0042, Dako). This has been prepared by conjugation of HRP to A0230 immunoglobulins and therefore has the same specificity.

### 2.3. Immunofluorescence

Confocal microscopy analysis (TCS DMIRE 2 Leica microscope, equipped with LCS Lite Software; Leica) was performed after incubating freshly fixed CSps and CDCs (4% paraformaldehyde, then permeabilized with 0.2% Triton X-100) with primary and secondary antibodies listed previously. Nuclei were stained by Topro3 nuclear dye (Invitrogen) [3]. Appropriate negative controls for autofluorescence and aspecific recognition by secondary antibodies have been performed during all staining protocols. Additionally, positive controls for all primary antibodies have been performed on human trophoblast preparations, as described in [27].

### 2.4. Sample Preparation and Heparin-Sepharose Affinity Chromatography

CMs from CSps (*n* = 20) and from CDCs (*n* = 20) were separately pooled and assayed by time-resolved immunofluorometric assay (TRIFA) before diafiltration on X-100A nonionic membranes (Diaflo, 43 mm diameter, Amicon) using a stirred cell (Model 52, Amicon); these CMs were finally concentrated to small volumes with 0.02 M Tris buffer solution, pH 7.2, 0.15 M NaCl. Affinity chromatography of concentrated CMs through Heparin-Sepharose (CL-6B, GE Healthcare) was performed as previously described [27]. Solutions containing 10 U of PAPP-A were applied to 1 × 6 cm column filled with the solid phase and equilibrated with the same Tris buffer solutions, and they allowed recycling at a flow rate of 25 mL/hour for 1 hour. One mL fractions were eluted with equilibrating buffer, followed by stepwise increase of NaCl concentration from 0.3 M to 0.9 M NaCl. Fractions were pooled on the basis of their absorbance at 278 nm and submitted to time-resolved immunofluorometric assay (TRIFA).

### 2.5. Immunofluorometric and Enzyme Immunoassays (EIA)

PAPP-A concentrations were measured in individual CSp- and CDC-CMs, in pools of CSp-CMs and pools of CDC-CMs and in their fractions, obtained from Heparin Sepharose chromatography, by TRIFA (DELFIA Xpress 6000, PerkinElmer), which recognizes dPAPP-A and PAPP-A/proMBP. Fluorescence of Europium at 615 nm was the endpoint of the automated assay, which uses pooled third trimester pregnancy serum-derived pregnancy-associated proteins WHO 78/610 reference preparation [30] as a Standard (WHO International Laboratory for Biological standards, Statens Serum Institute, Copenhagen, Denmark). Assay sensitivity was 5 mU/L. 

PAPP-A concentrations were also assessed by direct sandwich EIAs on 96-well plates (Nunc-Immuno Plate) based on three different capture antibodies: (a) 4PD4 (0.14 *μ*g/100 *μ*L), (b) A0230 (0.1 *μ*g/100 *μ*L), and (c) 5H9 (0.09 *μ*g/100 *μ*L). Antibodies, diluted in PBS, were used for coating, which was performed at 4–8°C for 12–18 hours. Wells were washed three times with PBS and nonspecific adsorption sites were blocked for 1 hour by 250 *μ*L of Casein Blocking Buffer (Thermo Scientific) at room temperature. After washing, 75 *μ*L sample and 25 *μ*L of fetal calf serum, or their dilutions in Blocking Buffer, were added to the wells and plates incubated for 18–24 hours at 4–8°C. After three washes, the same detection antibody (P0042, 1 : 400 in the same buffer solution) was added to the three immunoassays, and incubation continued at 37°C for 1 hour. After five more washes with 250 *μ*L blocking buffer, colour was developed by allowing 100 *μ*L of tetramethyl benzidine (ImmunoPure TMB substrate kit, Thermo Scientific) to react with HRP for 15 minutes at room temperature. Reaction was stopped with 100 *μ*L of 1 M H_2_SO_4_ and absorbance was measured at 450 nm. The combinations of the three capture antibodies with P0042 for detection were intended to assay dPAPP-A, dPAPP-A and PAPP-A/proMBP, and PAPP-A/proMBP, respectively. 

The two pools of CMs, previously calibrated by TRIFA against WHO 78/610 RP, were individually tested at different dilutions by the three EIAs available. Serial dilutions of the same WHO RP were also used as positive controls in the A0230- and 5H9-based EIAs or negative controls in the 4PD4-based EIA. Sensitivities of the EIAs were 20 mU/L (for 4PD4), 5 mU/L (for A0230), and 40 mU/L (5H9).

### 2.6. Statistical Analysis

Data are presented as mean value ± standard error of the mean. Significance of difference between any two groups was determined by two-sided Students *t*-test, and a final value of *P* < 0.05 was considered significant. 

## 3. Results

**Figure 1:**
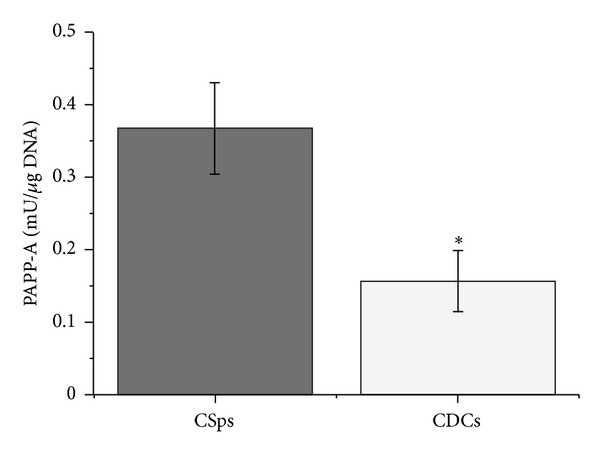
Quantification of PAPP-A secretion in CSps and CDCs conditioned media. PAPP-A concentrations were measured by immunofluorometric assay in individual 96-hour incubation CMs, randomly selected for CSps (*n* = 6) and CDCs (*n* = 5), and normalised to the total DNA content of the culture. PAPP-A normalised levels were significantly higher in CSp-CMs than in CDC-CMs (*P* = 0.018).

PAPP-A concentrations were initially assessed on individual CMs (*n* = 40) by TRIFA, which recognizes both homodimeric PAPP-A and PAPPA/proMBP complex. Interestingly, the obtained concentrations were biologically significant, being in the same order of magnitude of those routinely measured by the clinical laboratory of our university in maternal sera screened for Down's syndrome risk at 9–12 weeks of pregnancy (400–1900 mIU/L) and much higher than those present in plasma of patients with myocardial infarction (9.2–46.6 mIU/L) [21]. When separately pooled, CSp-CMs and CDC-CMs contained 810 mU/L 78/610 RP (range 220–1280 mU/L) and 1510 mU/L 78/610 RP (range 390–3010 mU/L) of PAPP-A, respectively. However, when normalized to the total DNA content of the culture, PAPP-A levels were significantly higher in CSp-CM versus CDC-CM, as shown in Figure 1. The presence of PAPP-A in pools of CSp-CMs and pools of CDC-CMs has been firstly assessed by affinity chromatography on heparin-sepharose column, a procedure known for its selectivity in recognizing PAPP-A and previously used for PAPP-A/proMBP separation from normal term maternal serum [25, 26, 31]. In these experiments more than 80% of the immunoassayable PAPP-A was eluted with 0.6 M NaCl, thus exhibiting a chromatography behavior similar to that shown by the proMBP-complexed enzyme. Recognition of dPAPP-A by Heparin was expected, since the binding of the enzyme to a similar cell surface glycosaminoglycan represents the prerequisite for addressing its proteolytic activity on the cell surface [30].

**Figure 2:**
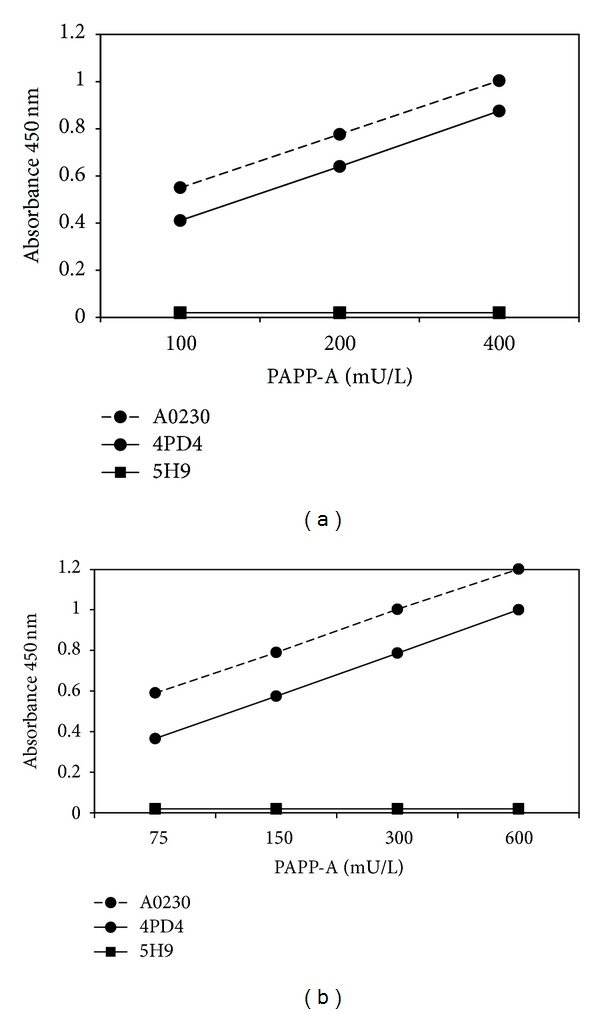
Immunoassayable PAPP-A using antibodies with different specificity. Titration of PAPP-A concentrations from CSp-CM (a) and CDC-CM (b) by three different EIAs. A0230 antibody (recognizing both dPAPP-A and PAPP-A/proMBP), 4PD4 antibody (specific for dPAPP-A), and 5H9 antibody (specific for the proMBP moiety) were used as capture antibodies for both CMs. In all cases, P0042 polyclonal antibody (HRP—conjugated anti-PAPP-A) was used for detection. The anti-proMBP/anti-PAPP-A antibody combination failed to recognize PAPP-A/proMBP complex in both CMs, whereas it was previously shown to be reactive with the pooled third trimester pregnancy serum-derived WHO 78/610 RP and with first and third trimester pregnancy human placental extracts [27].

Characterization of secreted PAPP-A in CMs was performed by EIA using capture antibodies of different specificity but with the same HRP-conjugated capture antibody. As shown in Figure 2, the monoclonal antibody specific for dPAPP-A (4PD4) was able to recognize the antigen in CMs from both CSps (Figure 2(a)) and CDCs (Figure 2(b)). The antibody reacting with dPAPP-A and PAPP-A/proMBP (A0230) also exhibited immunoreactivity for the two CMs. By contrast, the proMBP subunit-specific antibody (5H9, used in combination with HRP-conjugated PAPP-A polyclonal antibody P0042) failed to react with the same media. On the other hand, capacity of the same antibody combination (5H9 and P0042) of reacting with PAPP-A/proMBP, present in pregnancy sera or placenta extracts, has been previously demonstrated [27] and confirmed by its ability of reacting with the WHO 78/610 RP at PAPP-A concentrations as low as 40 mU/L. Taken together, these results indicate that only active dPAPP-A and not PAPP-A/proMBP is secreted in both CSp-CM and CDC-CM.

**Figure 3:**
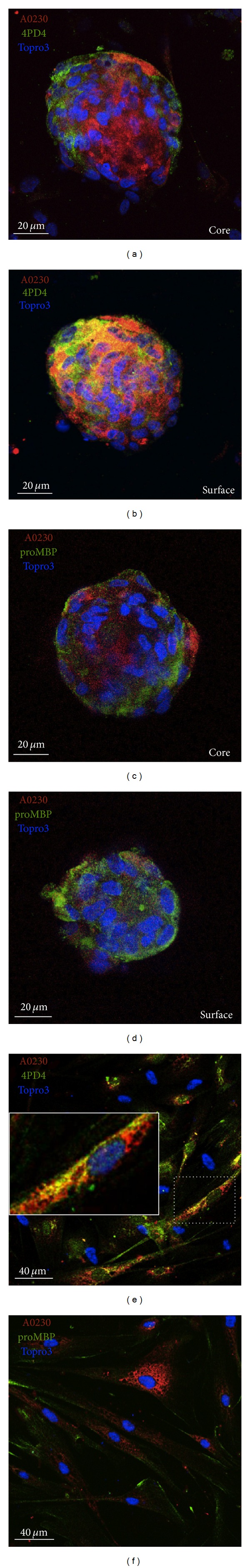
Immunofluorescence and confocal analysis of PAPP-A in CSps and CDCs. Representative confocal images for the detection of PAPP-A in CSps (a–d) and CDCs (e-f) by immunofluorescence: PAPP-A/proMBP complex and free PAPP-A are stained by A0230; homodimeric PAPP-A is recognized by 4PD4 antibody and pro-MBP by 5H9. Note that (a–d) display different focal planes (core or surface) of the same CSp, showing how the spatial distribution of dPAPP-A and proMBP, respectively, varies inside the 3D structure of the CSp. A higher magnification panel of the corresponding evidenced area is shown as insert in (e).

Immunofluorescence confocal analyses revealed that staining patterns obtained with the antibody against both dPAPP-A and PAPP-A/proMBP (A0230) partially overlapped with those of anti-dPAPP-A antibody (4PD4), suggesting a large extent of recognition of the same epitope (Figures 3(a) and 3(b)).

Immunocytochemical control stainings on human placental trophoblast preparations revealed the ability of all primary antibodies (A0230, 4PD4, 5H9) to detect PAPP-A, confirming their specificity (data not shown). Furthermore, negative controls showed no unspecific fluorescence of our secondary antibodies (data not shown). Based on these results, the uncomplete overlapping of the two antibodies A0230 and 4PD4 might be due to the different accessibility of their specific epitopes.

However, as evidenced by confocal images acquired at different focus planes, in 3D CSps, the homodimeric active PAPP-A seemed preferentially associated with outer cell layers (Figure 3(b)), usually housing the more differentiated cells [3, 32], consistently with the model of the CSp as an *in vitro* niche-like structure of spatially regulated gene expression and functions [6, 32, 33]. In a similar manner, the overlapped stainings of A0230 and 5H9 could be due to cross-reactivity of the first with proMBP (Figures 3(c) and 3(d)). When CDCs were stained with 4PD4 antibody, dPAPP-A appeared localized near the membrane and superimposed, at least in part, to the staining given by A0230 (Figure 3(e)). The presence of proMBP within CSps and CDCs was proven by the positive patterns shown by 5H9 antibody (Figures 3(d) and 3(f)). The same patterns did not allow distinguishing the free from the complexed proMBP, given the specificity of the antibody used for the subunit of proMBP. Direct immunocytochemical lines of evidence of the PAPP-A complex formation on the cell surface could not be obtained, since an antibody specific for the complex is not available. 

## 4. Discussion

IGF1 and the regulation of its availability play important roles in cardiac pathophysiology [34]. PAPP-A is one of the better characterized systems for the control of IGF1/IGFBPs binding and the consequent modulation of IGF1 bioavailability and activity. In this study, we detected significant amounts of secreted active PAPP-A in CPC-CMs. In particular, CSps secreted higher PAPP-A concentrations than CDCs in their respective CMs (Figure 1). CSps have a characteristic three-dimensional structure representing an *in vitro* niche-like microenvironment. This distinctive 3D conformation improves the paracrine potential of CSps versus CDCs, as previously described [5]. Given that it has been already shown that CSps produce higher levels of IGF1 and IGFBPs compared to CDCs [5, 32], we can speculate that the higher production of PAPP-A could be necessary for the dynamic regulation of the IGF axis, supporting the notion of an important role for the IGF1-network in CPC biology. Interestingly, our immunoassays show that only the active homodimeric PAPP-A form is secreted by CSps and CDCs (Figure 2). The data from immunofluorescence analysis confirm the intracellular presence of active dPAPP-A (Figure 3) and a preferential distribution in the CSp external layers. This is consistent with the notion of the CSp as a finely regulated niche-like structure [6, 32, 33], where the activity and availability of regulatory proteins, such as IGF1, must be under spatial and biological control. This is also consistent with the evidence of the binding capacity of the newly synthesized PAPP-A on the cell surface, where it posttranslationally modulates IGFBPs by limited proteolysis [30].

However, we were unable to ascertain the presence of PAPP-A/proMBP complex on the cell surface by immunofluorescence confocal microscopy. This failure could be due to the lack of specificity of the primary antibodies used for targeting the complex only, since A0230 antibody recognized epitopes present in the PAPP-A subunit and in the proMBP subunit, while 5H9 antibody is not able to distinguish free from bound proMBP. Lack of identification of the PAPP-A/proMBP complex might be due also to the high rate of formation and detachment of the complex from the cell surface, to its very weak capacity of surface binding [35], or to a substantial change in the balance between complexed and uncomplexed PAPP-A. Failure of the proMBP/PAPP-A-based sandwich immunoassay to detect the complex secreted into the CMs, together with the assessment of the expression of proMBP by cells, seems to support the last hypothesis. Perhaps, the multistep procedure of the complex formation involving a disulphide interchange between the two proteins might be altered in our experimental conditions. A number of experimental procedures are known to be able to affect the dynamics of the postsynthetic interaction of proMBP with its target proteins at the cell surface. They include regulatory mechanisms as redox potential, presence of reductase, metals, sulfhydryl compounds, and mutations of cysteine residues in proMBP, involved in the reduction and formation of disulphide bonds participating in the complex formation [36]. Failure of proMBP to complex with PAPP-A might represent a cellular strategy to control IGFs activity. Indeed, recently it has been demonstrated that disulphide cleavage in the extracellular domains of cell surface receptors, as well as in secreted soluble proteins, may be considered a switch for protein function [37]. 

The specificity of the monoclonal antibody 4PD4 used in this study to detect dPAPP-A has been previously tested by other authors using sandwich immunoassay [38]. Its capacity to recognize dPAPP-A without cross-reactivity with PAPP-A/proMBP complex is confirmed in our study, by using it as the capture antibody in combination with the HRP-conjugated anti-PAPP-A immunoglobulin as the detection antibody. Since this enzymatic immunoassay was unable to react with a pool of term pregnancy sera and with the WHO 78/610 RP, probably the sensitivity of the assay (20 mU/L) did not allow the detection of the small fraction of dPAPP-A (less than 1%) at the concentration tested, whereas the same antibody was able to recognize dPAPP-A in conditioned media from CDCs and CSps, allowing the clear identification of the antigen as dPAPP-A.

In conclusion, we have firstly demonstrated that free dimeric PAPP-A is expressed and released by CPCs in the form of CSps and CDCs at biological and clinical physiological relevant concentrations, being of the same order of magnitude as the circulating level found in peripheral blood of late first trimester pregnancy and ACS patients. Given the crucial cardioprotective and prosurvival roles of IGFs, both autocrine and paracrine, for the first time, our data demonstrate the production of active dimeric PAPP-A, besides IGFs and IGFBPs [5], by CSps and CDCs. It can be consistently speculated that PAPP-A is part of the IGFs network and that it participates in IGFs bioavailability and bioactivity on CPCs and their microenvironment. Furthermore, based on their potent PAPP-A secretory profile, a possible contribution of CPCs to elevated PAPP-A serum levels occurring in ACS could be speculated as a consequence of spontaneous, albeit limited, activation of endogenous heart regeneration in response to injury. Detection of PAPP-A secreted by the cells into their culture media depends, at least in part, on its molecular structure and it is dependent on the specificity of those antibodies recognizing dimeric PAPP-A, which can successfully be used for assaying this CPC-derived protease. Finally, CPCs seem to represent a novel highlyproductive source of dPAPP-A protein, which may be used for analytical and biochemical purposes, such as assay standards and/or antigen purification for specific antibodies productions.

## References

[1] Bach LA (2004). The insulin-like growth factor system: towards clinical applications. *The Clinical Biochemist Reviews*.

[2] Mahmoudabady M, Mathieu M, Touihri K (2009). Cardiac insulin-like growth factor-1 and cyclins gene expression in canine models of ischemic or overpacing cardiomyopathy. *BMC Cardiovascular Disorders*.

[3] Messina E, De Angelis L, Frati G (2004). Isolation and expansion of adult cardiac stem cells from human and murine heart. *Circulation Research*.

[4] Smith RR, Barile L, Cho HC (2007). Regenerative potential of cardiosphere-derived cells expanded from percutaneous endomyocardial biopsy specimens. *Circulation*.

[5] Chimenti I, Smith RR, Li T-S (2010). Relative roles of direct regeneration versus paracrine effects of human cardiosphere-derived cells transplanted into infarcted mice. *Circulation Research*.

[6] Chimenti I, Forte E, Angelini F, Giacomello A, Messina E (2012). From ontogenesis to regeneration: learning how to instruct adult cardiac progenitor cells. *Progress in Molecular Biology and Translational Science*.

[7] Gaetani R, Barile L, Forte E (2009). New perspectives to repair a broken heart. *Cardiovascular and Hematological Agents in Medicinal Chemistry*.

[8] Makkar RR, Smith RR, Cheng K (2012). Intracoronary cardiosphere-derived cells for heart regeneration after myocardial infarction (CADUCEUS): a prospective, randomised phase 1 trial. *The Lancet*.

[9] Chimenti I, Rizzitelli G, Gaetani R (2011). Human cardiosphere-seeded gelatin and collagen scaffolds as cardiogenic engineered bioconstructs. *Biomaterials*.

[10] Gaetani R, Rizzitelli G, Chimenti I (2010). Cardiospheres and tissue engineering for myocardial regeneration: potential for clinical application. *Journal of Cellular and Molecular Medicine*.

[11] Takeda N, Manabe I, Uchino Y (2010). Cardiac fibroblasts are essential for the adaptive response of the murine heart to pressure overload. *Journal of Clinical Investigation*.

[12] Bendall SC, Stewart MH, Menendez P (2007). IGF and FGF cooperatively establish the regulatory stem cell niche of pluripotent human cells in vitro. *Nature*.

[13] Lawrence JB, Oxvig C, Overgaard MT (1999). The insulin-like growth factor (IGF)-dependent IGF binding protein-4 protease secreted by human fibroblasts is pregnancy-associated plasma protein-A. *Proceedings of the National Academy of Sciences of the United States of America*.

[14] Laursen LS, Overgaard MT, Soe R (2001). Pregnancy-associated plasma protein-A (PAPP-A) cleaves insulin-like growth factor binding protein (IGFBP)-5 independent of IGF: implications for the mechanism of IGFBP-4 proteolysis by PAPP-A. *FEBS Letters*.

[15] Kumar A, Mohan S, Newton J (2005). Pregnancy-associated plasma protein-A regulates myoblast proliferation and differentiation through an insulin-like growth factor-dependent mechanism. *Journal of Biological Chemistry*.

[16] Chen B-K, Leiferman KM, Pittelkow MR, Overgaard MT, Oxvig C, Conover CA (2003). Localization and regulation of pregnancy-associated plasma protein A expression in healing human skin. *Journal of Clinical Endocrinology and Metabolism*.

[17] Brambati B, MacIntosh MCM, Teisner B (1993). Low maternal serum levels of pregnancy associated plasma protein A (PAPP-A) in the first trimester in association with abnormal fetal karyotype. *British Journal of Obstetrics and Gynaecology*.

[18] Meloni P, D’Angeli I, Piazze J (2009). First trimester PAPP-A levels associated with early prediction of pregnancy induced hypertension PAPP-A to detect gestational hypertension. *Hypertension in Pregnancy*.

[19] Boldt HB, Conover CA (2007). Pregnancy-associated plasma protein-A (PAPP-A): a local regulator of IGF bioavailability through cleavage of IGFBPs. *Growth Hormone and IGF Research*.

[20] Oxvig C, Sand O, Kristensen T, Gleich GJ, Sottrup-Jensen L (1993). Circulating human pregnancy-associated plasma protein-A is disulfide-bridged to the proform of eosinophil major basic protein. *Journal of Biological Chemistry*.

[21] Bayes-Genis A, Conover CA, Overgaard MT (2001). Pregnancy-associated plasma protein A as a marker of acute coronary syndromes. *New England Journal of Medicine*.

[22] Bayes-Genis A, Conover CA, Schwartz RS (2000). The insulin-like growth factor axis: a review of atherosclerosis and restenosis. *Circulation Research*.

[23] Funayama A, Shishido T, Netsu S (2011). Serum pregnancy-associated plasma protein A in patients with heart failure. *Journal of Cardiac Failure*.

[24] Schulz O, Reinicke M, Krämer J (2011). Pregnancy-associated plasma protein A values in patients with stable cardiovascular disease: use of a new monoclonal antibody-based assay. *Clinica Chimica Acta*.

[25] Iversen KK, Teisner AS, Teisner B (2008). Pregnancy Associated Plasma Protein A, a Novel, Quick, and Sensitive Marker in ST-Elevation Myocardial Infarction. *American Journal of Cardiology*.

[26] Conti E, Carrozza C, Capoluongo E (2004). Insulin-like growth factor-1 as a vascular protective factor. *Circulation*.

[27] D’Elia P, Marzioni D, Castellucci M, Moccia C, Pala A (2012). Homodimeric pregnancy-associated plasma protein-A in normal human placenta of first and third trimester of pregnancy: biochemical and morphological observations. *Placenta*.

[28] Chimenti I, Gaetani R, Barile L (2012). Isolation and expansion of adult cardiac stem/progenitor cells in the form of cardiospheres from human cardiac biopsies and murine hearts. *Methods in Molecular Biology*.

[29] Pala A, Di Ruzza A, Curtilli L (1987). Interference of an *α*2 component in immunological determinations of pregnancy-specific *β*1 glycoprotein in serum. *Clinica Chimica Acta*.

[30] Glerup S, Kløverpris S, Laursen LS (2007). Cell surface detachment of pregnancy-associated plasma protein-A requires the formation of intermolecular proteinase-inhibitor disulfide bonds and glycosaminoglycan covalently bound to the inhibitor. *Journal of Biological Chemistry*.

[31] Suzuki Y, Takada J, Isaka K, Takayama M, Grudzinskas JG (1997). Isolation of pregnancy-associated plasma protein A. *Placenta*.

[32] Li T-S, Cheng K, Lee S-T (2010). Cardiospheres recapitulate a niche-like microenvironment rich in stemness and cell-matrix interactions, rationalizing their enhanced functional potency for myocardial repair. *Stem Cells*.

[33] Forte E, Miraldi F, Chimenti I (2012). TGF*β*-dependent epithelial-to-mesenchymal transition is required to generate cardiospheres from human adult heart biopsies. *Stem Cells and Development*.

[34] Abbas A, Grant PJ, Kearney MT (2008). Role of IGF-1 in glucose regulation and cardiovascular disease. *Expert Review of Cardiovascular Therapy*.

[35] Laursen LS, Overgaard MT, Weyer K (2002). Cell surface targeting of pregnancy-associated plasma protein A proteolytic activity: reversible adhesion is mediated by two neighboring short consensus repeats. *Journal of Biological Chemistry*.

[36] Qin Q-P, Kokkala S, Lund J, Tamm N, Voipio-Pulkki L-M, Pettersson K (2005). Molecular distinction of circulating pregnancy-associated plasma protein A in myocardial infarction and pregnancy. *Clinical Chemistry*.

[37] Hogg PJ (2003). Disulfide bonds as switches for protein function. *Trends in Biochemical Sciences*.

[38] Serebryanaya DV, Tamm NN, Koshkina EV, Katrukha AG Immunochemical properties of PAPP-A isolated from human atherosclerotic coronary arteries.

